# A Genetic Make Up of Italian Lipizzan Horse Through Uniparental Markers to Preserve Historical Pedigrees

**DOI:** 10.3390/biology13121087

**Published:** 2024-12-23

**Authors:** Alessandra Crisà, Irene Cardinali, Andrea Giontella, Maurizio Silvestrelli, Hovirag Lancioni, Luca Buttazzoni

**Affiliations:** 1 Consiglio per la Ricerca in Agricoltura e l’Analisi dell’Economia Agraria (CREA), Research Centre for Animal Production and Aquaculture, Via Salaria 31, 00015 Monterotondo, Italy; luca.buttazzoni@crea.gov.it; 2 Department of Chemistry, Biology and Biotechnology, University of Perugia, Via Elce di Sotto 8, 06123 Perugia, Italy; cardinali_irene@libero.it; 3 Centro di Studio del Cavallo Sportivo, Dipartimento di Medicina Veterinaria, Università di Perugia, Via S. Costanzo, 4, 06126 Perugia, Italy; andrea.giontella@unipg.it (A.G.); maurizio.silvestrelli@unipg.it (M.S.)

**Keywords:** Lipizzan breed, mtDNA, maternal lines, haplotype, haplogroup, MSY, pedigree

## Abstract

Lipizzan is a famous horse breed dating back to 1580 when the original stud of Lipica was established by the Hasburg Archduke Charles II. Currently, the Italian State Stud of Lipizzan Horses (ASCAL) is a conservation nucleus managed through strict mating rules where mitochondrial DNA sequences are used to verify the correct assignment of mares to a historical pedigree maternal lineage. Here, we analyzed the D-loop sequences of Lipizzan horses from the ASCAL in Monterotondo (Rome, Italy) in order to confirm their pedigree assignment to known female founder families. The concurrent investigation of the paternal counterpart based on variation in the male-specific region of the Y chromosome (MSY) showed a prevalence of HT02 (80%), typical of the Neapolitan/Oriental wave. The mtDNA polymorphisms identified shaped nine haplotypes that were unequivocally assigned to each of nine classical mare families of the stud (Africa, Almerina, Argentina, Deflorata, Djebrin, Fistula, Ivanka, Sardinia, Spadiglia), while the Europa and Theodorosta families shared a tenth haplotype. New polymorphisms were identified in a not previously studied region (np 16100–16350). The mtDNA phylogenetic analysis revealed that the Lipizzans of the Monterotondo stud belong to six haplogroups (B, C, G, L, M, Q), out of the 18 recorded for the equine species. This work enabled us to identify and preserve ten haplotypes from the historical maternal lines in a small stud kept in genetic segregation for over 100 years.

## Simple Summary

The Lipizzan is a famous horse breed dating back to 1580 when the original stud of Lipica was established by the Hasburg Archduke Charles II. The stud was intended to supply the court in Graz with riding and light carriage horses. Nowadays, several State Studs derived from the Austrian–Hungarian Empire with slightly differentiated breeding goals are present in eight European Countries. The Lipizzan world population is small, gene flows are limited, and the breed is at risk of genetic erosion. Currently, the Italian State Stud of Lipizzan Horses (ASCAL) is a conservation nucleus managed through strict mating rules where mitochondrial DNA (mtDNA) sequences are used to verify the correct assignment of mares to a historical pedigree maternal lineage. In order to preserve the genetic variability of the Lipizzan horse breed, we performed mtDNA analyses focused on maintaining all pedigree maternal lines (eleven) in the stud, and an evaluation of the variability in the male-specific region of the Y chromosome (MSY), which revealed a Neapolitan/Oriental origin of Lipizzan stallions.

## 1. Introduction

The Lipizzan breed originated in 1580, when the Habsburg Archduke Charles the Second was the ruler of Inner Austria, with the city of Graz as the capital [1], after the tripartition of the kingdom of Austria stated by his father, the Emperor Ferdinand the First, in 1564 [2]. The Archduke needed riding and light carriage horses of Spanish origin for his court [3], so he established his own stud in Lipica (Italian: Lipizza; German: Lippiza) [4,5]. The history of the breed started in 1581 with 6 stallions and 24 mares imported from Spain, but pedigree records only trace the breed back to the mid-1700s, when the current breed standard and the name of the breed itself began to set [6,7,8,9]. Currently, the base population of Lipizzan horses is divided into a number of state-owned studs, with limited exchange of horses over the last few decades. The breeding goals of the studs are changing over time and across different State stud. Whereas the primary goal of the Austrian stud at Piber is still to provide horses for classic dressage at the Spanish Riding School in Vienna, the Hungarian stud in Szilvásvárad has specialized in breeding top horses for coach driving. The Slovenian, Slovakian, and Croatian studs are breeding riding horses, while the Romanian studs are providing stallions for improving the local farm horse population [10].

The Italian State Stud of Lipizzan horses is located in Monterotondo, near Rome, and it breeds horses that are direct descendants of those kept in Lipizza before World War I (WWI), shared between Austria and Italy in 1919, when 109 horses were given to Italy, seized by the German Army in 1943, and partly returned to Italy by the U.S. Army in 1947 (80 horses and their breeding records). In 1955, the stud was transferred to the Ministry of Agriculture and Forestry, which committed its Institute of Animal Research to maintaining the nucleus of purebred Lipizzans through traditional mating strategies. Free natural mating is the traditional method, performed by mating groups based on the historical male and female lines maintained since the Habsburg Empire [11]. The nucleus comprises all six of the classical male lines (Conversano, Neapolitano, Pluto, Favory, Maestoso, and Siglavy) and 11 of the classical female families (Africa, Almerina, Argentina, Deflorata, Djebrin, Europa, Fistula, Ivanka, Sardinia, Spadiglia, and Theodorosta), representing the genetic heritage of the Habsburg Empire [11]. From the beginning of the XVIII Century, the rulers of Austrian Imperial studs adopted naming conventions based on “male lines” and “female families”. Stallions were identified by the name of the founder of their male line followed by the name of their mother, and dams were given a name chosen among a definite pool of names assigned to their female lineage. This naming scheme had the function of avoiding matings between close relatives [11] while preserving the founders’ heritage. Although the Lipizzan International Federation recognizes 17 classical female families [12], the original book kept in Italy since 1919 states that the mares Bradamanta (1777) and Capriola (1785) were, respectively, daughters of Deflorata (1767) and Europa (1774). This suggests that Bradamanta and Capriola should not be considered independent families and there are only 15 classical female families.

Line and family assignations rely on pedigree data, but these data are prone to errors. Mitochondrial DNA (mtDNA) sequencing has been extensively used to study intra- and inter-species genetic relationships and structures since it is maternally inherited, haploid, and not subject to genetic recombination [13]. In particular, the displacement loop (D-loop) hyper-variable region (HVR) of mitochondrial DNA yields the most information in matrilineal studies since it possesses a high degree of sequence variation but only a moderate mutation rate [14]. The mtDNA control region in horses contains two highly variable segments (HVR1 and HVR2), four conserved blocks (CSB), and variable eight base pair repeats [15,16]. MtDNA was used to study the domestication and genetic diversity of modern horse breeds and it is also useful to detect pedigree errors ([17], as an example). When different mtDNA haplotypes appear among members of the same maternal family, pedigree errors are likely to have occurred. Some authors characterized the mtDNA control region in the Lipizzan horse breed and identified a total of 45 different haplotypes [6,18,19,20,21].

Recently, many efforts have been made to also produce Y chromosomal DNA data through the analysis of sire lines, deepening the molecular phylogeny of the main horse breeds worldwide and giving important insights into their breeding history (as reviewed in [22]). In Italian horse populations, three haplotypes of the male-specific region of the Y chromosome (MSY) were found out of the six retrieved in Europe [23], with different frequencies: HT1 (the ancestral haplotype, 35%), HT2 (Neapolitan/Oriental wave, 51%) and HT3 (Thoroughbred wave, 14%) [22,24]. These data suggested a selective pressure on local horse breeds operated through specific breeding schemes and focused on the refinement stocks.

The principal aim of the present study was to provide genetic insights into the pedigree of the Italian Lipizzan Horse from both the maternal and paternal sides. The molecular characterization of the mtDNA D-loop in the Italian nucleus of Lipizzan horses enabled us to verify family assignations based on pedigrees and could possibly allow us to assign a unique mtDNA haplotype to each maternal family. Moreover, a longer region was sequenced to uncover new polymorphisms and to define putative private mtDNA lineages. Concomitantly, the evaluation of the male counterpart through the analysis of the MSY haplotypes in Lipizzan stallions will ensure successful stock management. Finally, to verify the evolution of inbreeding in the closed nucleus after culling mares with undesired mtDNA haplotypes, a few statistical analyses on pedigree were performed and compared to older estimates [11].

## 2. Materials and Methods

Almost all the horses in the Monterotondo Stud represent the progeny of stallions and mares kept in Lipizza before the First World War (WWI) and given to Italy on 17 July 1919. The exceptions are a stallion given in 1919, the son of a stallion from Lipizza from the Stud of Earl Eltz in Vukovar; two stallions bought from the Kingdom of Yugoslavia in the 1930s; and and few mares exchanged with the Stud of Piber with a pedigree entirely tracing back to mares and stallions from Lipizza.

Data from the original handwritten Studbooks were recorded in a database: the oldest ancestor was a mare born in 1738 and lacking parental information, while the most recent ancestor was a mare born in 1900. Since the year 1900, the Stud has been kept in complete genetic segregation.

Blood samples were collected during veterinary controls from a total of 157 animals in 2014, 2016, and 2023. Then, DNA was extracted by using the Wizard Genomic DNA kit (Promega Corporation, Madison, WI, USA) following the manufacturer’s instructions. PCR amplification in an initial study started about 10 years ago was performed as previously described by [18,25]. In more recent analyses, two different primer pairs were designed for the upstream (UP-FW: ACCATCAACACCCAAAGCTG, nucleotide position (np) 15425–15444; UP-RW: TTTCCTATGTCCCGCTACCA, np 16125–16106) and downstream (DOWN-FW: ATTCAGTCCATGGTAGCGGG, np 16096–16115; DOWN-RW: GGCTGAGCAAGGTGTTATGAG, np 216–206) regions. A touchdown protocol was used with an annealing temperature of 59 °C. The PCR products were purified and sent to EUROFINS Genomics (Ebersberg, Germany) for sequencing on both strands. MtDNA sequences were aligned to the horse sequence JN398377 [26] (identical in the mitochondrial control region to the old reference sequence X79547) using MUSCLE in MEGA 11.0 software [27], and haplotypes of the Monterotondo horses were defined by detected nucleotide variation. Phylogenetic relationships were inferred through the Maximum Likelihood method and Hasegawa–Kishino–Yano plus gamma model [28] in MEGA 11.0 using a bootstrap value of 1000 to assess confidence in the branching order. The evolutionary relationships among maternal family haplotypes and haplogroup classification defined by [26] were analyzed through the construction of a median-joining network using Network 10.2 [29], accessed on 3 September 2024.

Three polymorphic sites of the MSY (YE3, YE17, and YXX) were analyzed on male samples as previously described [23], and the data were compared to those already reported in [24].

Finally, statistics on pedigree were performed. Starting from living horses with no progeny, the total number of founders and ancestors, equivalent founders (f_e_), and equivalent ancestors (f_a_) were computed by the program PRORIG, of the package PEDIG [30]. Inbreeding was computed by the program VANRAD and the effective number of founder genomes (N_g_) was computed by the program SEGREG from the same package.

## 3. Results

Most maternal diversity studies of horses are based on sequencing of the upstream mtDNA D-loop (HVR1, 15440-16100) [25,31,32,33]. Refs. [6,18] described further variants in Lipizzan horses in the downstream D-loop (HVR2, 16351-16600).

Ten years ago, our first attempt at studying maternal diversity involved analyzing the UP and DOWN regions with already reported primers of 93 females from the ASCAL. The alignment of mtDNA sequences allowed for the definition of 10 diverse haplotypes that were compared with those already identified by [6,18] and were named in the same way considering the sequence homology. Table 1 shows the distribution of horses across eleven pedigree families (rows) and ten different mtDNA haplotypes (columns) found in 2014. Each cell of the table reports the number of sequenced females for the combination pedigree family/haplotype.

Table 1 gives evidence that only the pedigree families—Djebrin (9 females) and Ivanka (15 females)—showed a unique haplotype (“Dubovina” and “J”, respectively), while all the other haplotypes were found in more than one family, with two pedigree families (Almerina and Argentina) carrying as many as four different haplotypes. The Capriola and J haplotypes were the most frequent in our stud, and Slavina and Wera the rarest.

Being aware that Century-old, handwritten pedigree registrations might well include unwanted mistakes, and that different haplotypes found in the same maternal line indicate pedigree errors, to maintain all pedigree families and all 10 of the haplotypes, in 2014, an empirical approach was taken to uniquely associate the detected haplotypes with the pedigree families of the mares. First of all, haplotypes found in unique pedigree female families (“Dubovina”, “Batosta”, “Slavina”, and “Wera”) were assigned to the corresponding families: Djebrin, Africa, Almerina, and Sardinia, respectively. The haplotype “Allegra” was assigned to Fistula because it was detected in 5 females of that family, while only two females carrying that haplotype had been regarded as belonging to the Deflorata family, and Almerina had already been uniquely associated with the haplotype “Slavina”. Haplotype “X” was assigned to Argentina, the family where it was most frequent (five females out of eight). The haplotype “Capriola” was assigned to the Deflorata family, because the other families where it appeared (Argentina, Fistula, Almerina, and Sardinia) had already been associated with other haplotypes. For the same reason, the “Monteaura” haplotype could only be assigned to the Spadiglia family, while the haplotype “U”, detected both in the Europa and Theodorosta families, each of them having only one mare in the stud, could not be uniquely assigned. While the mare belonging to the pedigree family Europa came from the Austrian State Stud of Piber, the oldest mare of the Theodorosta family, definitely carrying the “U” haplotype, was born in Monterotondo in 1981. Only the 55 females carrying the haplotype assigned to their pedigree family were retained in the stud, while all of the other females were sold.

**Figure 1:**
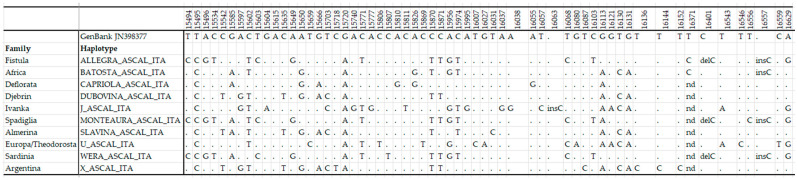
Polymorphisms among the ten family haplotypes within the mtDNA control region in comparison with the sequence JN398377 for *Equus caballus.* The dot indicates correspondence with the reference sequence; “nd” indicates that the sequence for that position is not available.

In 2023, we proceeded with a verification of the current stud by using the new primers; thus, on the DNA of 32 mares, we were able to obtain more complete sequences of the mtDNA D-loop region between the UP and DOWN regions. Identical sequences were joined to the same haplotype/family, and the sequences of the haplotype were deposited in GenBank with accession numbers PP495263-67, PP495269-72, PP534165. We retained the name given initially by [6] and added a suffix to indicate that they are specific to the animals in the Monterotondo stud. The complete sequence of the D-loop region has been identified for the Allegra_ASCAL_ITA and Batosta_ASCAL_ITA haplotypes, while for the other haplotypes, we do not have coverage from 16320 to 16399 as the maximum and 16360 to 16394 as the minimum. The alignment of our sequences with the sequence JN398377 from nucleotide position (np) 15445 to np 16660 allowed for the identification of the mutational motifs reported in Figure 1.

**Figure 2:**
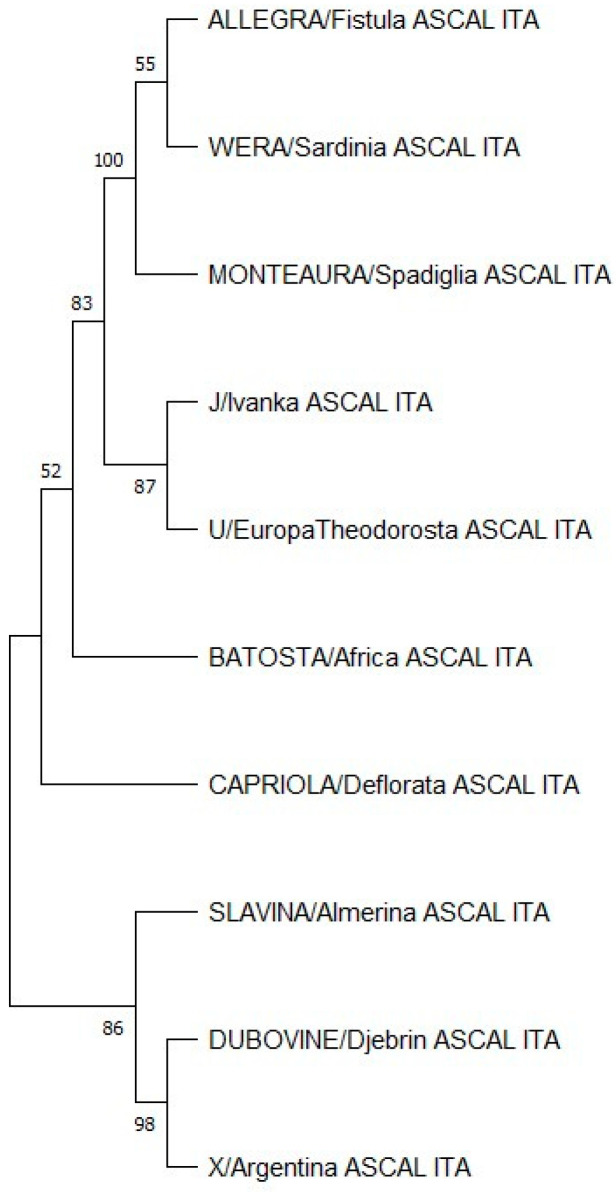
Phylogenetic relationships among the haplotypes/families present in the Monterotondo stud in 2023. Branches corresponding to partitions reproduced in less than 50% of bootstrap replicates are collapsed. Next to the branches are shown the percentages of replicate trees in which the associated taxa clustered together in the 1000 bootstrap test.

The relationships among the haplotypes of the maternal line inferred by MEGA 11 are represented in Figure 2. Higher bootstrapping values were identified in the divergence of the (1) Fistula ASCAL/Sardinia ASCAL families from Spadiglia ASCAL; (2) Fistula ASCAL/Sardinia ASCAL/Spadiglia ASCAL form Ivanka ASCAL and Europa/Theodorosta ASCAL; (3) Almerina ASCAL from Djebrin ASCAL/Argentina ASCAL; and (4) Djebrin ASCAL from Argentina ASCAL.

With the aim of returning a more complete dataset of samples collected from the ASCAL (Monterotondo) during the years, we also included the Lipizzans published in [25]. On a total of 157 animals, we verified that the assignment of each haplotype to a specific maternal family observed in 2014 was confirmed, and we proceeded with a more complete haplogroup assignment and evolutionary phylogenetic analysis.

**Figure 3:**
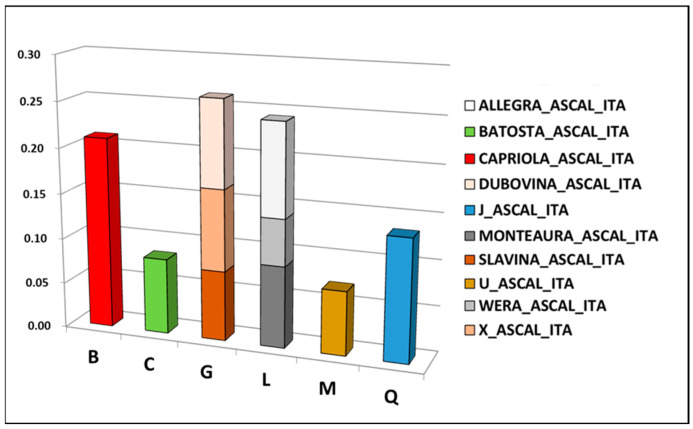
Frequency distribution of ten Lipizzan haplotypes within each mtDNA haplogroup.

A total of six mtDNA haplogroups were identified. The most frequent was the haplogroup G (26%), represented by three haplotypes (U_ASCAL_ITA, X_ASCAL_ITA, SLAVINA_ASCAL_ITA), followed by L (24%; ALLEGRA_ASCAL_ITA, MONTEAURA_ASCAL_ITA), B (21%; HT08), Q (13%; J_ASCAL_ITA), C (8%; BATOSTA_ASCAL_ITA), and M (7%; U_ASCAL_ITA) (Figure 3 and Table S1).

**Figure 4:**
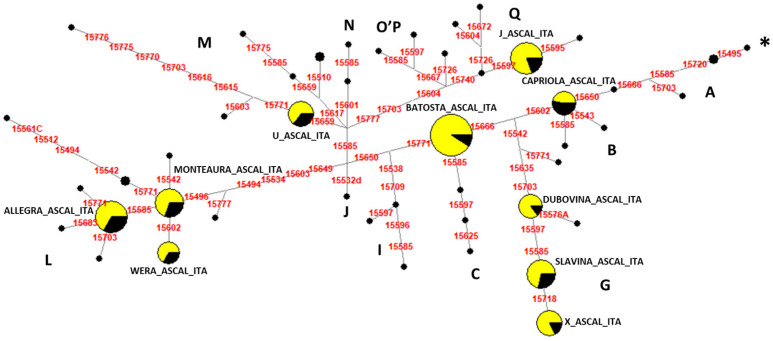
The median-joining network based on the control-region sequences of all Lipizzan horses from the ASCAL (*N* = 157, highlighted in yellow) and GenBank (*N* = 90, black). * indicates a haplotype identical to equine sequence X79547. Each single-nucleotide polymorphism (SNP) indicates a single mutational event; each circle is proportional to the number of samples carrying the same haplotype, and haplogroups are indicated with capital letters.

The median-joining network analysis based on the control-region sequences of 157 Lipizzan samples from the ASCAL and 90 sequences recorded in GenBank [6,18,31,33,34] showed the evolutionary relationships among all the haplotypes retrieved in the Lipizzan breed (Figure 4).

**Figure 5:**
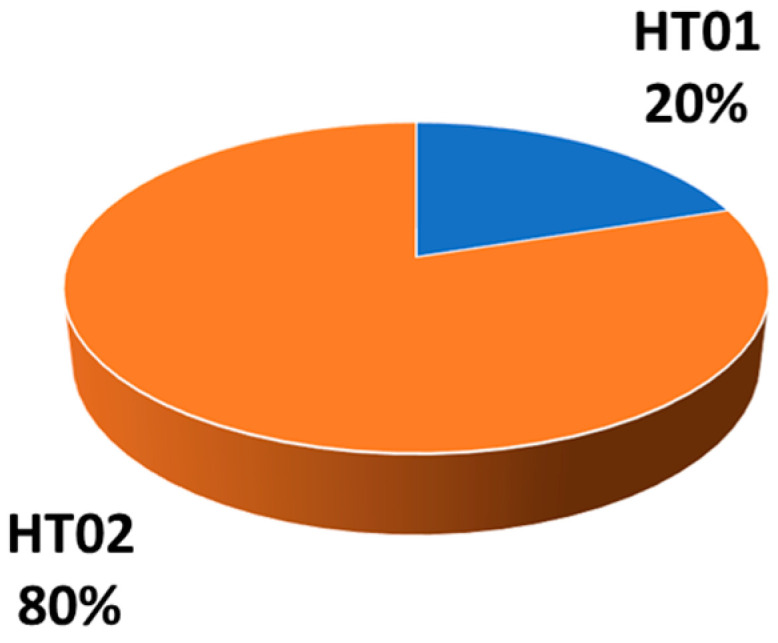
MSY haplotype frequencies of Lipizzan stallions from ASCAL in Monterotondo (Italy).

The evaluation of the male counterpart consisted of sequencing of the three MSY loci (YE3, YE17, and YXX) and subsequent alignment to the references JX646942.1, JX646950.1, and JX647030.1. Eight Lipizzan stallions revealed the presence of the mutation in the polymorphic site YE17, nucleotide position 1277, thus representing haplotype HT02 [23]. No other variations were detected; therefore, the remaining samples had the haplotype HT01. Overall, the most frequent haplotype was HT02, reaching a frequency of 80% (Figure 5).

The Monterotondo stud is a closed farm, kept in genetic segregation for over 120 years, with very complete pedigree data spanning over 23 generations. The ratio between horses with both parents known and the total number of horses (pedigree completeness) is 81.6%. We wanted to ascertain how the inbreeding level had been affected after culling mares carrying unwanted haplotypes. Therefore, we re-evaluated some pedigree statistics computed 16 years ago [11]. In addition to culling, about two generations had passed, and some corrections of ancient pedigree data had to be performed. The results of the analysis, tracing back from active stallions and mares in 2023, are reported in Table 2.

## 4. Discussion

Nowadays, genetic characterization is recognized as crucial in conservation programs. The evaluation of all existing lineages from both maternal and paternal sides is a pre-condition for breeding strategy and, thus, for the protection and balanced development of breeds. Due to its high mutation rate and its strictly maternal inheritance, mtDNA sequencing has become a widely used tool for tracing genetic diversity among horse breeds and pedigree populations in Europe [6,18,34,35,36,37,38] and especially in local breeds from Italy [25,31,33,39].

In this study, the sequence analysis of the mtDNA control region of the ASCAL mares identified ten haplotypes that were arbitrarily assigned after a step-by-step process to each of the 11 maternal classical families, although it was not possible to differentiate the mtDNA haplotypes of the Europa and Theodorosta families. The decision to associate each haplotype with a maternal pedigree family was taken to make it easier to maintain all the haplotypes. Nevertheless, the association is completely valid only within the Monterotondo stud.

Due to the maternal inheritance of mtDNA, one can expect that all animals belonging to the same maternal lines will have the same mtDNA haplotype. However, during the verification carried out in 2014, we found 38 females not correctly assigned to the pedigree maternal family. Some differences were also found among our matchings between mtDNA haplotypes and pedigree families and the study in [40] on European State Studs. While the mtDNA haplotypes we found for seven classical families (Deflorata, Fistula, Spadiglia, Djebrin, Africa, Almerina, Europa) were in agreement with the findings by [18,40], differences were found for four classical pedigree families (Argentina, Ivanka, Sardinia, Theodorosta). No females of the latter four families showed the “X” haplotype [40], which was found in most samples (5) of the Argentina family in Monterotondo. Then, almost all Monterotondo females belonging to the family Ivanka (or Famosa) carried the haplotype “J”, which [18] found only in two studs: Piber (Austria) and Monterotondo (Italy). The Ivanka family was first assigned the haplotype “Strana” [6], while the later haplotype “Strana” was associated with the Croatian family Munja, and the Ivanka family was no longer considered [40]. Ref. [40] associated the Sardinia family with the haplotype “Betalka” (not found in Monterotondo). However, Sardinia was the only family in Monterotondo carrying the haplotype “Wera”, and we accepted the association Sardinia–Wera to preserve this haplotype. It should be noticed that “Wera” belongs to the group of haplotypes coming from Iberian and North African horses, possibly from the actual foundation of the breed [6], as the original founder mare Sardinia, born in 1776. Finally, the Theodorosta family was represented in Monterotondo by a unique mare carrying the “U” haplotype, the same as that of the Europa family. Overall, among all our Lipizzans, the haplotype with the highest frequency was Capriola, found in 38 sequences out of 157 (24%), followed by J (15%) and Allegra (13%).

One of the goals of the present study was to verify whether further variants could be found in longer mtDNA sequence fragments. The authors of [35] showed that increasing the length of the analyzed mtDNA control region in Arabian horse populations may improve the degree of informativeness.

The complete sequence of the D-loop region has been identified for the Allegra_ASCAL_ITA and Batosta_ASCAL_ITA haplotypes, while for the other haplotypes, we do not have coverage from 16320 to 16399 as the maximum and 16360 to 16394 as the minimum.

In our study, the UP control region between the end *tRNA^Phe^* and the first bases of the repeat region (np 15445-16153) showed high sequence variability with 55 polymorphic sites among the 10 haplotypes, while the DOWN control region (np 16154-16660) showed a lower degree of sequence variability with only 8 polymorphic sites.

Compared to the mt SNPs reported by [18,19], we identified 25 new polymorphisms between the 15827 and 16152 positions between almost one of the ten haplotypes and the sequence JN398377. Ref. [25] sequenced the region from 15491 to 16100 in 10 Italian horse breeds, including Lipizzan. In this region, polymorphisms were identified until np 16100. Even though our analyses of the Lipizzan mtDNA control region showed that the fragment UP control region was sufficient to discriminate among classical female families in Monterotondo [25,40], we here report 10 more SNPs among the ASCAL mtDNA haplotypes.

The Allegra_ASCAL_ITA (Fistula) and Wera_ASCAL_ITA (Sardinia) haplotypes had identical sequences in the DOWN region, as did the groups Capriola_ASCAL_ITA (Deflorata), Slavina_ASCAL_ITA (Almerina), Dubovina_ASCAL_ITA (Djebrin), and X_ASCAL_ITA (Argentina), which could suggest the existence of a common maternal ancestor. This hypothesis is in agreement with the likely Spanish origin of the founder mares of these families. These groups were already evidenced in [40], except for the X haplotype.

The dendrogram obtained with the family haplotypes showed some groups whose relationships reflect what was previously identified by the study of [40]. In particular, Slavina/Dubovina/X/Batosta/Capriola are included in the C1 cluster, and Allegra/Wera/Monteaura are included in the C4 cluster. The J haplotype is included in the C2a cluster and the U haplotype in the C3a cluster.

The geographical origin of maternal lines, an indicator of molecular divergence time, was estimated by [26] based on the entire mtDNA sequences of 83 horse breeds that were classified into 18 haplogroups. The authors concluded that the proposed classification could be used in the study of the remains of ancient horses, phylogenetic relations of modern breeds, and intra-breed diversity. Following the same approach and the haplogroup classification nomenclature, we analyzed the Lipizzan dataset and found that Monterotondo females belong to six mitochondrial DNA haplogroups: B, present in Europe and the Middle East; C, recorded only in the Middle East with low frequencies; G, from Central Asia and the Middle East; L and M, both typical of European horses; Q, mainly distributed in Asia and the Middle East. In particular, the network analysis highlighted that only two haplogroups are represented by different haplotypes: G, carried by Almerina (Slavina_ASCAL_ITA), Argentina (X_ASCAL_ITA), and Djebrin (Dubovina_ASCAL_ITA) families, and L, retrieved in Spadiglia (Monteaura_ASCAL_ITA), Sardinia (Wera_ASCAL_ITA), and Fistula (Allegra_ASCAL_ITA)This suggests a common maternal origin for these families. The same results could also be observed in the dendrogram obtained with the family mtDNA haplotypes, since the two groups showed a close relationship between these maternal lines. The other B, C, M, and Q haplogroups were represented by a single haplotype each, carried by Deflorata (Capriola_ASCAL_ITA), Africa (Batosta_ASCAL_ITA), Europa and Theodorosta (U_ASCAL_ITA) and Ivanka (J_ASCAL_ITA), respectively.

The origins of the haplogroups fit well the known history of the Lipizzan breed, which was built over centuries by mating mares and stallions coming from Spain, Italy, Denmark, other Imperial Studs in Central Europe, and finally, the Middle East. The only exception is represented by haplogroup Q, here associated with the ancient family Ivanka, which originated in 1754 in the stud of Koptchan (now Kopčany in the Slovak Republic). It is possible that this haplotype derived from horses used by the Mongolian light cavalry in their invasions of Europe in the XIII Century [41,42] or from later Turkish invasions of Hungary from the XV to the XVII Century [43].

A similar Oriental origin was also found in Lipizzan stallions from Monterotondo, where the high frequency of HT02 (80%, the same frequency as previously reported [24]) suggests a Neapolitan/Oriental origin. Similar distributions were found in Maremmano (80%), an Italian local breed, descending from the Etruscan native horses [24], and Giara (75%), a feral native breed from Sardinia, probably brought to the island by the Phoenicians [38]. Identical haplotypes were also recorded in other Lipizzan stallions, but at different frequencies, namely HT01 = 33% and HT02 = 67% [29,44]. This distribution of MSY haplotypes is the result of historical breeding practices: due to the intensive selection of stallions, a few sire lines were established, and HT02 typically dominates the Lipizzan horse breed, especially in the Monterotondo stud.

The comparison of inbreeding levels between 2007 and 2023 shows a strong reduction in genetic variability, with average inbreeding growing up to 19.53%. However, the average inbreeding of the 32 matings in the breeding season 2023 was 0.196, with a minimum of 0.174 and a maximum of 0.221. Examining the marginal contributions of actual ascendants, it was found that 31 ascendants (14 stallions born from 1911 to 1998, and 17 mares born from 1931 to 2000, all from classical lines or families) were able to explain all the variability in the stud: the overall marginal contribution from them was 81.91% for stallions and 18.09% from mares.

The completeness of the pedigree (the most recent mare with unknown parents was born in the year 1900) and its depth (up to 23 generations) make these analyses quite reliable.

Due to the long genetic segregation, and to the sharp reduction in the number of animals following the exclusion of females carrying the “incorrect” haplotype, inbreeding grew to nearly 20%. This is a very high level even if compared to the value of 13.9 of the Thoroughbred population [45], but no obvious adverse effect has been noticed so far. However, while average inbreeding grew up to 20%, the number of effective ascendants only diminished by 2.4%, attesting the random distribution of females carrying the “incorrect” haplotype and the correctness of the current mating scheme.

Further investigation will focus on whole-genome data in order to bring the genomic characterization of the breed to the maximum level of resolution.

## 5. Conclusions

Genetic variations in both uniparental markers were investigated in order to verify genealogical tracing and pedigree relationships in the Italian Lipizzan Horse.

This study confirmed the assignment of a unique haplotype to ten out of eleven female pedigree families kept in the Monterotondo stud, thus confirming D-loop mtDNA as a powerful marker to analyze the genetic diversity of maternal lines in Lipizzans and verifying the overall reliability of pedigree records spanning over two centuries. The upstream mtDNA control region proved to be sufficient to differentiate among the families kept in Monterotondo. Based on these results, the Monterotondo stud has kept only mares carrying the mtDNA haplotype assigned to their pedigree family, so it is now possible to assign any female from the Monterotondo stud to a specific maternal family, regardless of possible recording mistakes. More importantly, maintaining all haplotypes in the stud will contribute to preserving genetic variability. On the other hand, the MSY analysis confirmed a Neapolitan/Oriental origin for almost all the Lipizzan stallions belonging to the stud. Furthermore, being the oldest horse breed in Europe and because of its historical connection to the Austro-Hungarian Empire, the Lipizzan horse is a living part of the European Cultural Heritage. Thus, in addition to biological reasons, there are historical and cultural needs for monitoring the conservation status of this breed: in December 2022, Lipizzan Horse Breeding Traditions was inscribed in the representative list of the UNESCO intangible cultural heritage of humanity. This inclusion not only acknowledges the breed’s historical significance, but also emphasizes the socio-cultural responsibility of preserving such genetic legacies. This dual biological and cultural approach can be globally used as a blueprint for the heritage conservation of other breeds.

## Figures and Tables

**Table 1:** The number of females of the Monterotondo stud in 2014 after matching the 10 mtDNA detected haplotypes (columns) with the classical pedigree families (rows). The frequencies of the 10 haplotypes in the stud are also reported. The chosen matches are highlighted.

Family	Haplotype										
	X	Capriola	Allegra	Monteaura	U	Dubovina	Batosta	J	Slavina	Wera	Total
Africa				2			7				9
Almerina		4	3						2		9
Argentina	5	4			3			1			13
Deflorata		6	2								8
Djebrin						9					9
Europa					1						1
Fistula		6	5								11
Ivanka								15			15
Sardinia		3		7						2	12
Spadiglia	3			2							5
Theodorosta					1						1
*N*° females	8	23	10	11	5	9	7	16	2	2	93
% haplotype	8.6	24	10.7	12.8	5.3	9.7	7.5	17.2	2.1	2.1	

**Table 2:** Inbreeding analysis of the Monterotondo stud horses.

	Year	
	2007	2023
Reference animals	53	65
Total founders	232	250
Total ascendants	1259	1290
Equivalent founders (f_e_)	47.4	47.6
Effective ascendants (f_a_)	12.02	11.72
f_e_/f_a_ (bottlenecks)	3.94	4.06
Founder genomes (N_g_)	2.60	2.39
f_g_/f_e_ (genetic drift)	0.0549	0.0502
Average inbreeding	16.2%	19.53%

## Data Availability

The original data presented in this study are openly available in GenBank with the accession numbers PP495263-67, PP495269-72, PP534165.

## Supplementary Materials

The following supporting information can be downloaded at: https://www.mdpi.com/article/10.3390/biology13121087/s1, Table S1: Control-region haplotypes and haplogroup classification of the mtDNAs of Lipizzan horses from Monterotondo.
